# The Mediating Role of Negative Affect in the Relationship Between Anger Rumination and Somatic Symptoms

**DOI:** 10.62641/aep.v54i4.2193

**Published:** 2026-08-15

**Authors:** Concepción Muñoz-Sosa, Bárbara Luque, Esperanza García-Sancho, Carmen Tabernero

**Affiliations:** ^1^Department of Social Psychology and Anthropology, University of Salamanca, 37007 Salamanca, Spain; ^2^Department of Psychology, University of Cordoba, 14071 Cordoba, Spain; ^3^Department of Personality, Evaluation and Psychological Treatment, University of Malaga, 29010 Malaga, Spain

**Keywords:** somatisation, rumination, negative affect, chronic pain, functional syndromes

## Abstract

**Background:**

A high prevalence of somatisation is associated with a strong impairment of quality of life. Recent research suggests common underlying mechanism of central sensitisation in functional syndromes and chronic pain, with emotion regulation (ER) processes closely linked to somatic symptoms. This study examines the relationship between negative affect (NA) and anger rumination (AR) and its influence on somatisation.

**Methods:**

Five hundred seven individuals participated (mean age = 34.60 ± 13.22 years; 380 women). They completed the Patient Health Questionnaire, Negative Affect Scale, and Displaced Aggression Questionnaire. A cross-sectional multivariate factorial relational design was used. Descriptive, multiple linear regression, and mediation analyses were conducted on the total and sex-separated samples.

**Results:**

Data analysis revealed a significant positive relationship between NA and AR (*r *= 0.55, *p* < 0.01), and both were significantly associated with somatisation (rumination: *r* = 0.37, *p* < 0.01; NA: *r *= 0.54, *p* < 0.01). Simple linear regressions confirmed that higher levels of NA and AR individually predicted increased somatic symptoms in both sexes. However, multiple regression analyses revealed that the effect of AR on somatisation was reduced in women and became non-significant in men when accounting for NA. Mediation analysis showed a significant indirect effect of AR on somatisation through negative affect (*B* = 0.005, 95% bootstrap confidence interval [0.004, 0.006]), accounting for 62.5% of the total effect. The mediation pattern was replicated in both women (indirect effect *B* = 0.005, 95% confidence interval [0.004, 0.007]) and men (indirect effect *B* = 0.004, 95% confidence interval [0.002, 0.006]). These findings indicated that part of the effect of AR on somatisation occurred under the influence of NA.

**Conclusions:**

NA and AR were associated with increased somatic symptoms. These findings suggest the benefit of developing interventions that encourage the use of functional ER strategies. These can help to reduce NA, which in a prolonged form can constitute a vulnerability for organisms, especially in those people with a tendency to somatise.

## Introduction

Somatisation includes functional complaints that lack structural organic pathology to justify them [1]. It involves the interaction of physiological, cognitive, and affective factors between the symptomatic and central nervous systems [2]. In Spain, the prevalence of somatisation in primary care is 20%, with a notable impact on quality of life, damage due to incessant medical testing, disabling symptoms, and a significant economic strain on patients, families, and society [3, 4]. A key controversy of this phenomenon is the boundary between mental and physical health that it inhabits [5]. Misunderstanding arises from equating bodily symptoms solely with organic causes, even though a high prevalence of symptoms lack identifiable medical disorders [6].

Recognition of psychological and social influences underscores the need to revise classical biomedical explanations of functional syndromes [7], although a persistent view is that only bodily dysfunction can produce ‘real’ symptoms [8]. This perspective coexists with critiques highlighting the absence of specific biomarkers, syndrome overlap, and the lack of universally accepted diagnostic criteria [9]. Proposals for a generic functional syndrome suggest a shared pathophysiology centred on central sensitisation: a process where the central nervous system amplifies nociceptive sensory input, perceived as discomfort or pain [10, 11]. This mechanism, observed in chronic pain, is also studied as a primary driver in functional and somatic syndromes [3, 10, 12].

Pain is the most frequent symptom and diagnostic criterion for somatisation [13]. Some somatic syndromes fall under the category of primary chronic pain, influenced by psychosocial stress, trauma, and intrapsychic conflicts [14]. Shared aetiological factors include neuroendocrine stress axis sensitisation [15]; limbic system hyper-reactivity [16]; and alterations in the endocrine, immune, and pain regulation systems [3].

Cognitive and emotional mechanisms contribute to the development of somatic symptoms [1]. Cognitions and emotions are closely related to neural pathways involved in pain regulation and peripheral processes including muscle tension, inflammation, and autonomic system dysregulation [14]. Evidence supports a bidirectional influence between emotion and pain in functional syndromes, which could clarify underlying psychopathological processes and reveal shared vulnerability factors [17, 18]. The comorbidity between somatisation and affective disorders does not undermine their independence [1]. There is evidence of differentiation between pathways, with pro-inflammatory immune processes being responsible for somatic symptoms [19].

Research indicates that affective processes play a role in chronic distress associated with somatic symptom [20]. In particular, deficits in affective processing and emotion regulation (ER) have been identified as risk factors for psychosomatic illnesses [21], partly due to their association with immunological disturbances [22]. Emotion dysregulation from somatosensory amplification affects autonomic, endocrine, and immune function. This increases vulnerability to inflammatory processes, which, in turn, increases the severity of somatic syndromes [23].

Initial classifications of affective processes distinguished between positive and negative dimensions. Negative affect (NA) is conceptualised as a higher-order affective dimension, referring to a general tendency to experience aversive emotional states including feelings such as sadness, anger, and anxiety. NA has been linked to biological system dysregulation that sustains functional symptoms [24], increases somatic and physical symptoms [25, 26], and plays a mediating role in ER, affecting somatic complaints [27]. NA influences processing style and the use of ER strategies [28], and evidence points to its interrelationship with repetitive negative thinking [29].

Continuing with this focus on affective processes, ER refers to the ability to modulate how emotions are experienced and expressed [30]. It may contribute to the development and treatment of somatic symptoms [1, 20]. In somatisation, researchers have proposed a disintegrated interaction between the cognitive and bodily components of ER [31]: while neurophysiological findings suggest deficits in central nervous system capacity, reflected in prefrontal and amygdalar activity [32], neurobiological studies have emphasised significant neural connectivity between emotional, somatosensory, and motor subsystems [33, 34].

Although NA has been widely studied as a general vulnerability factor, specific negative emotions may play distinct roles in somatic symptomatology. Among these, anger has received comparatively less attention, despite its potential relevance [35]. Anger comprises physiological, cognitive, and behavioural elements [36], and shares neurophysiological pathways with pain [3]. Its dysregulation contributes to psychological, affective, personality, and health issues [37]. Studies indicate that anger increases the severity of somatic symptoms by disrupting pain modulation systems [38], thereby contributing to symptom intensity and interference [39, 40].

In this context, anger rumination (AR) or perseverative cognition has been identified as a maladaptive ER strategy characterised by repetitive and persistent negative thinking (i.e., NA) [41, 42]. This process contributes to the maintenance of negative emotional states and sustained physiological activation, allowing psychosocial factors to affect bodily functioning [43]. Research suggests that the accumulation of negative and recurrent thoughts triggers psychological and physiological responses, leading to mental health issues, vulnerability to depression and anxiety, distress, functional impairment, and somatic discomfort [44, 45]. Rumination is associated with elevated cortisol levels; altered immune responses; and heightened cardiovascular, endocrine, immune, and neurovisceral activity [43].

Consistently, researchers have reported that patients with a chronic somatic condition have a premorbid tendency to ruminate [46], and there is a bidirectional relationship between rumination and pain severity [47]. In chronic pain, rumination mediates the relationship between pain intensity and catastrophising tendencies [48]. Recent studies have shown that heightened AR in women with fibromyalgia exacerbates pain and symptom severity [38, 49]. These findings reinforce rumination as a risk factor for the development and maintenance of various disorders [50].

Although the present study focuses specifically on AR, NA was included as a broader affective dimension to capture general emotional vulnerability. NA reflects a general tendency to experience aversive emotional states, whereas AR represents a more specific cognitive-emotional process.

It is also important to consider that differences arising from cultural factors and gender roles may, in turn, influence the strategies and resources used in emotional regulation [51]. Research indicates that while women frequently engage in rumination processes – often linked to depression and internalised anger-men tend towards avoidance, suppression, and externalised expression [52, 53, 54]. This difference may reflect the influence of gender-related factors, understood as a socioculturally constructed and multidimensional concept encompassing roles, behaviours, and norms [53], which plays a crucial role in shaping both the experience and expression of distress. In this context, recent evidence has highlighted the limited availability of high-quality research addressing gender differences and public stigma related to persistent somatic symptoms, as well as the risk of misinterpretation or minimisation of these symptoms in women [55]. Therefore, it is essential to examine gender differences in the relationship between NA, AR, and somatisation within a biopsychosocial and gender-informed framework to better understand these processes.

**Fig. 1.**
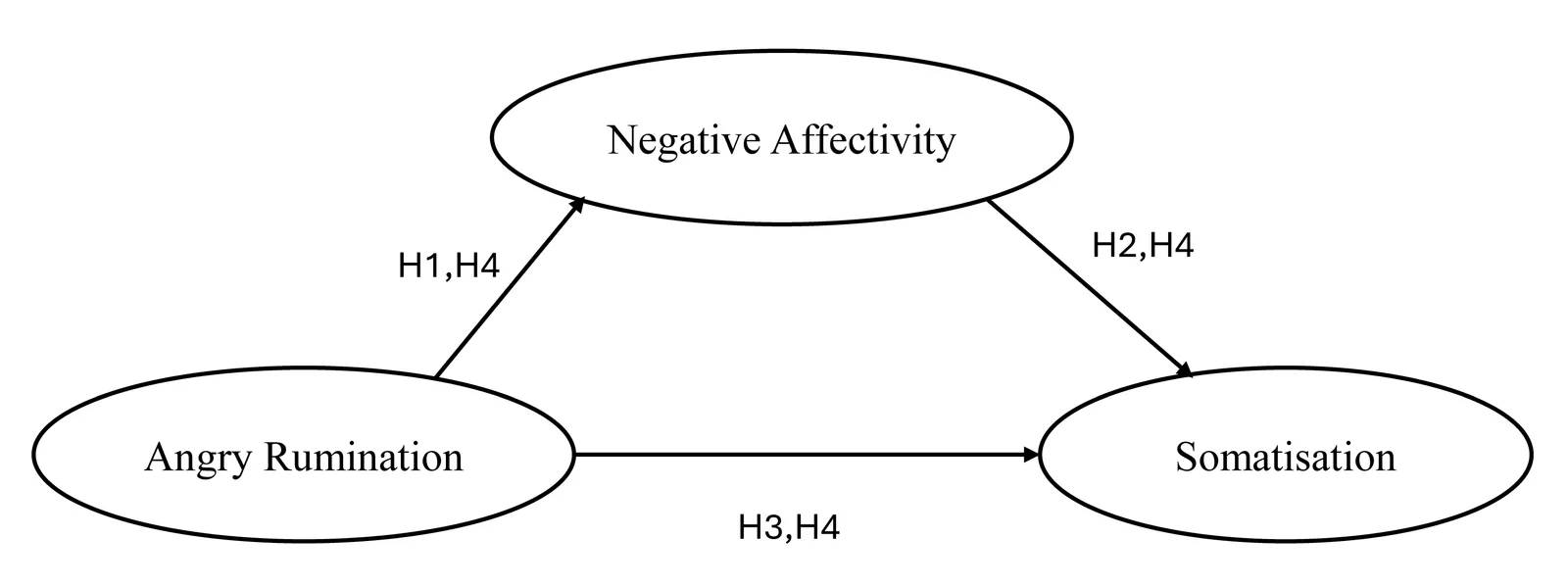
**Hypotheses and mediation analysis between observed variables**.

In summary, considering the relationships between NA and ER, particularly in relation to AR and somatisation, the main objective of this research was to provide evidence of the significance and direction of these relationships and to explore potential gender differences. This led to the following hypotheses (see Fig. 1):

H1. AR has a positive relationship with NA.

H2. Participants with greater NA show greater somatic symptomatology.

H3. Higher levels of AR lead to greater somatisation.

H4. NA mediates the relationship between AR and somatisation.

H5. Differences in the use of ER strategies between men and women influence somatic symptoms.

## Materials and Methods

### Design and Participants

Participants were recruited in May 2022 through chain sampling for voluntary and anonymous participation [56]. Confidentiality was ensured, and participants were informed about the research objectives and provided their consent to participate freely. This study adhered to the Declaration of Helsinki and received approval from the Córdoba Research Ethics Committee (date and version of the protocol: 1–6 January 2020; committee reference: 5166; report no. 327; approved on 28 September 2021).

A cross-sectional multivariate factorial relational design was employed without a control group. Data collection included demographic information and administration of scales to measure somatisation, AR, and NA.

The inclusion criteria specified healthy participants aged 18–60 years of Spanish nationality. The exclusion criteria were being under 18 or over 60 years of age, having a nationality other than Spanish, or failing to complete all the assessment scales. The initial sample comprised 584 individuals from the general population; 18 individuals of different nationalities and 59 participants who did not complete all scales were excluded. The final sample included 507 participants, of whom 380 were women and 127 were men. Complete demographic characteristics and mean scores are presented in Table 1.

### Measures

#### Patient Health Questionnaire (PHQ-15)

This study used the PHQ-15, originally validated by Kroenke [57] and by Iterian [58] for the Spanish population. The PHQ-15 assesses 90% of the most prevalent physical complaints in somatisation and has proven effective in both clinical practice and research in primary care [59]. Participants rate items based on symptom severity (0 = not experiencing, 1 = somewhat, 2 = a lot). The total score ranges from 0 to 30. The original scale demonstrated high reliability (α = 0.80), comparable to that of the Spanish version (α = 0.79) and the current study (α = 0.81).

#### Negative Affect

NA was measured with a reduced version of the Positive and Negative Affect Schedule [60] adapted for the Spanish population [61]. This scale measures the extent to which an individual experiences aversive emotional states. This version includes five items (scale: 0–4, range: 1–5) to assess negative emotional states, ranging from 1 (very slightly) to 5 (extremely). The total score for the NA subscale is obtained by summing the scores of the five items, with a total range of 5–25 points. Higher scores indicate a greater presence of NA. The NA subscale has shown good internal consistency, with Cronbach’s alpha ranging from 0.84 to 0.87 in the original validation study [61]. In this study, internal consistency was analysed to determine reliability, yielding Cronbach’s alpha of 0.90.

#### Rumination

The Displaced Aggression Questionnaire (DAQ) validated in Spanish by García-Sancho [62]. This scale assesses personality differences in the tendency to displace aggression and comprises three dimensions: affective (AR), cognitive (revenge planning), and behavioural (displaced aggression). The present study focused exclusively on the AR subscale, which consists of 10 items that assess the tendency to focus attention on angry moods and recall past anger experiences. Participants rate each item on a 7-point Likert scale, ranging from 1 (extremely uncharacteristic of me) to 7 (extremely characteristic of me). The total score for this subscale is calculated as the sum of the 10 items, with a total score range of 10–70 points. Higher scores indicate a greater tendency to experience AR. The Spanish version has a good internal consistency, with Cronbach’s alpha ranging from 0.69 to 0.83. The present study focused on AR, with Cronbach’s alpha of 0.94.

### Statistical Analyses

Statistical analyses were conducted with SPSS Statistics (Version 30.0; IBM Corp., Armonk, NY, USA), supplemented by the PROCESS macro for mediation analyses (Hayes). Descriptive statistics of socio-demographic variables were computed. Normally distributed continuous variables are described using means (*M*s) and *SD*s, and categorical variables are summarised using frequencies and percentages. Independent samples t-tests were conducted to examine gender differences in study variables. Homogeneity of variances was assessed using Levene’s test, and the appropriate t-test statistics were interpreted accordingly when the assumption of equal variances was not. Bivariate associations between variables were examined using Spearman’s rank correlation coefficients. Subsequently, linear and multiple regression analyses were performed to explore associations between NA and AR and interactions with somatisation, both in the total sample and stratified by sex (multi-group analysis). Prior to conducting regression analyses, assumptions of multicollinearity, independence of residuals, and model adequacy were evaluated. Multicollinearity was assessed using variance inflation factors (VIFs), while independence of residuals was examined using the Durbin–Watson statistic. In all operations, the difference of one item in the female-specific PHQ-15 questionnaire (menstrual pain) was considered.

To test H4, related to the mediating effect of NA, mediation analysis was performed using PROCESS (Model 4), with NA specified as the mediator. Indirect effects were estimated using bootstrapping with 10,000 resamples, and statistical inference was based on 95% bias-corrected confidence intervals (CIs).

## Results

### Subject Characteristics

First, descriptive analyses were conducted to characterise the study sample and examine potential sex differences across the study variables. Table 1 presents the socio-demographic characteristics and descriptive statistics of the sample.

**Table 1.** **Descriptive statistics for the sample**.

Characteristics		Total sample (N = 507)
Age (years)		34.60 ± 13.22
Sex		
	Women	380 (75%)
	Men	127 (25%)
Education level		
	Primary education	6 (1.2%)
	Secondary education	57 (11.2%)
	Vocational training (intermediate level)	12 (2.4%)
	Vocational training (advanced level)	45 (8.9%)
	University degree	196 (38.6%)
	Postgraduate	189 (37.3%)
	Another	2 (0.4%)

### Spearman Correlations

Mean scores were analysed by sex and age. Women (*n* = 380) had higher somatisation (*M *= 0.61, *SD* = 0.30) than men (*n* = 127; *M* = 0.45, *SD* = 0.27). Student’s *t*-test showed a significant difference between sexes, *t *(505) = 5.53, *p* < 0.001, indicating higher somatisation in women. There were no significant differences for the other variables. Spearman correlations were calculated for the total sample and separately for women and men (Table 2).

**Table 2.** **Means, standard deviations, and correlations between variables according to sex**.

Sample	Variables	Rumination	Negative affect	*T (df 505)*	*M*	*SD*
Total sample	Rumination	.		0.30	37.00	14.56
	Negative affect	0.55^**^	-	1.57	12.03	8.34
	Somatisation	0.37^**^	0.54^**^	5.53**	0.56	0.30
Women	Rumination	.		-	37.00	14.10
	Negative affect	0.55^**^	-	-	12.36	8.24
	Somatisation	0.38^**^	0.55^**^	-	0.61	0.30
Men	Rumination	-		-	36.55	15.92
	Negative affect	0.56^**^	-	-	11.02	8.58
	Somatisation	0.38^**^	0.51^**^	-	0.45	0.27

*Note:* **p < 0.01, *p < 0.05. *M*, mean; *SD*, standard deviation (*n* = 380 women, *n* = 127 men).

Data analysis revealed significant relationships between NA and somatisation for both sexes. In the total sample, AR was positively associated with NA and somatisation. Individuals with higher NA showed greater rumination of AR and vice versa, supporting H1. Those with higher NA also reported greater somatic symptoms, providing support for H2. In addition, there was a significant association between AR and somatisation, supporting H3, with greater AR corresponding to higher somatic symptomatology.

### Linear Regression

Simple linear regression analyses were conducted on the full sample to test the study hypotheses. Subsequently, gender-segmented analyses were performed (multi-group), where the sample of women included one additional item related to the symptoms of menstruation. The results were consistent across both sexes (Table 3).

**Table 3.** **Simple linear regression testing the study hypotheses for the total sample and by gender**.

Hypothesis and Group		*N*	*F (df1,df2)*	*R adj* ^2^	β
(H1) AR → NA					
	Total sample	507	217.38 (1, 505)	0.30	0.55***
	Women	380	154.96 (1, 378)	0.29	0.54***
	Men	127	61.86 (1, 125)	0.33	0.58***
(H2) NA → Somatisation					
	Total sample	507	180.93 (1, 505)	0.26	0.52***
	Women	380	155.28 (1, 378)	0.30	0.54***
	Men	127	40.99 (1, 125)	0.28	0.50***
(H3) AR → Somatisation					
	Total sample	507	87.46 (1, 505)	0.15	0.39***
	Women	380	71.49 (1, 378)	0.16	0.40***
	Men	127	23.52 (1, 125)	0.16	0.40***

*Note*: ***p < 0.001. AR, anger rumination; NA, negative affect; H1, anger rumination predicting negative affect. H2, negative affect predicting somatisation; H3, anger rumination predicting somatisation. β = standardised regression coefficient.

To examine the overall relationship between the variables, we conducted multiple linear regression with somatisation as the dependent variable and NA and AR as predictors (Table 4).

**Table 4.** **Multiple linear regression testing the study hypotheses for the total sample and by gender**.

	Total sample (*n* = 507)				
Predictors Variables	*F(df1, df2)*	*Model p*	*Radj* ^2^	β	*Predictor p*
NA (H2)	97.42 (2, 504)	0.001	0.28	0.44	<0.001
AR (H3)				0.15	<0.001
	Women (*n* = 380)				
NA (H2)	83.73 (2, 377)	0.001	0.31	0.46	<0.001
AR (H3)				0.15	0.003
	Men (*n* = 127)				
NA (H2)	22.44 (2, 124)	0.001	0.26	0.40	<0.001
AR (H3)				0.17	0.078

*Note*: AR, anger rumination; NA, negative affect; H1, anger rumination predicting negative affect. H2, negative affect predicting somatisation; H3, anger rumination predicting somatisation. β = standardised regression coefficient.

The effect of AR on somatisation decreased in women when accounting for NA. In men, this effect became nonsignificant. Including NA in the mediation model reduced the direct path of AR, suggesting partial mediation by NA.

### Mediation Effect

To test H4, we performed a simple mediation analysis on the total sample to examine whether NA mediated the relationship between AR and somatisation. AR had a significant total effect on somatisation, *B* = 0.008, *t *= 9.35, *p* < 0.001, 95% CI [0.006, 0.010]. The direct effect of AR on somatisation remained significant, *B* = 0.003,* t* = 3.24, *p* = 0.001, 95% CI [0.001, 0.005]. The indirect effect mediated by NA in the total sample was significant, *B* = 0.005; *p* < 0.001, 95% bootstrap CI [0.004, 0.006], indicating partial mediation. Statistical significance was determined by whether the CI excluded zero. The mediation proportion (indirect/total effect) indicated that 62.5% of the total effect was explained via NA. These findings support H4: NA appears to mediate a substantial portion of the relationship between AR and somatisation.

Finally, we conducted multi-group mediation analyses to examine potential gender differences (Table 5). Including NA as a mediator reduced the direct effect of AR on somatisation in women, though it remained significant. In men, this effect became nonsignificant. The bootstrap CI for the indirect association did not include zero, indicating that NA partially mediated the relationship between AR and somatisation in both sexes.

**Table 5.** **Mediation effects stratified by gender**.

Effects	Women (n = 380)			95% CI	Men (n = 127)			95% CI
	*B*	*t*	*p*		*B*	*t*	*p*	
Total effect	0.009	8.46	<0.001	[0.007, 0.011]	0.007	4.85	<0.001	[0.004, 0.010]
Direct effect	0.003	2.99	0.003	[0.001, 0.005]	0.003	1.78	0.077	[–0.001, 0.006]
Indirect effect	0.005	-	-	[0.004, 0.007]	0.004	-	-	[0.002, 0.006]

*Note*: CI, confidence interval.

## Discussion

Our aim was to investigate the relationship between NA, AR, and somatisation. We identified a directional relationship between AR and NA (supporting H1), reinforcing previous research [63]. Rumination fosters negative mood-congruent thinking (NA) [41, 42], maintaining arousal that affects the organism [43]. In turn, previous research has demonstrated that NA may have both direct and mediating effects on processing styles (ER) [64], fostering the use of dysfunctional strategies [28]. In the present study, however, we focused on the mediating role of NA in the relationship between AR and somatisation. This distinction allows us to integrate the broader theoretical association between NA and ER with the specific mediation model supported by our findings. NA increases vulnerability to both psychological and somatic processes [65] and intensifies rumination by heightening access to negative thoughts and emotions [66, 67].

Individuals with higher NA reported more somatic complaints, supporting H2 and consistent with previous findings [25, 26] indicating that the predominance of emotions like sadness or anger (i.e., NA) could pose a health risk [68]. NA can induce or amplify continuous physiological arousal and hypervigilance, leading to pain sensitisation and amplification [50]. It impacts immune and endocrine responses and, over time, contributes to chronic inflammation [69]. In this regard, NA is associated with the dysregulation of biological stress systems, reduced pain tolerance, somatic symptom maintenance, and overall mental and physical health issues [24, 70].

AR also predicted greater somatisation (supporting H3), underlining its influence on the development of somatic complaints and distress [43, 46]. Anger triggers muscle reactivity associated with symptoms and influences pain development and maintenance, enhancing pain perception and sensitivity [53], leading to more frequent and severe somatic symptoms [71, 72]. Anger dysregulation is implicated in health problems [51] and can exacerbate pain frequency and severity [39, 40]. The effect of AR on somatisation aligns with the impact of rumination on different psychological and somatic conditions [46, 47, 73]. Sustained emotional intensity can be detrimental to health because it generates an imbalance in the organism’s homeostasis that ends up causing discomfort, pain, and illness [74]. This chronic psychophysiological activity is linked to persistent sympathetic activation [38] and heightened vulnerability to somatic disorders [41].

In support of H4, NA mediated the relationship between rumination and somatic complaints. This finding is consistent with previous studies that have shown NA mediates emotion dysregulation processes, including avoidance and maladaptive coping strategies [75], as well as pain catastrophising [76]. Research on ER has shown that prolonged negative emotional states are linked to increased symptom reporting, physical health problems, and maladaptive immune responses [29, 74]. This finding is in line with previous literature suggesting that rumination contributes to the maintenance and intensification of negative emotional states, thereby increasing levels of NA [77]. Moreover, previous studies have reported associations between psychosomatic complaints and ER when controlling for NA [27, 28], as well as between rumination and post-traumatic grief symptoms in women [67, 69]. These findings emphasise that a predisposition to specific affective states and dysfunctional strategies can impact somatic health.

Evidence-based interventions such as cognitive-behavioural therapy [78, 79] and acceptance and commitment therapy [80] have demonstrated effectiveness in reducing maladaptive repetitive thinking, improving ER, and decreasing NA. Consistently, primary care intervention targeting ER strategies have shown reductions in anxiety and somatic symptoms, with decreased rumination [81].

Regarding H5, there was a higher prevalence of somatic symptoms in women, in line with prior studies [82, 83]. This finding may reflect differences in emotional processing and regulation shaped by both biological and sociocultural factors. Neurobiological differences in the processing and modulation of somatic stimuli [82] as well as variations in biological stress responses [84] and increased pain sensitivity [85] have been proposed as contributing factors. These processes may be influenced by NA and emotions like anger, which are associated with stress reactivity [86, 87].

A substantial body of research has shown gender differences in ER strategies, with women more likely to engage in ruminative processes and men tending to rely on avoidant or suppressive strategies [88]. Rumination has been associated with sustained NA and prolonged physiological activation, which may increase sensitivity to bodily sensations and contribute to the development and maintenance of somatic symptoms [89].

In the present study, AR predicted greater somatisation in men when NA was not considered. In this regard, it is worth considering the factors that may influence the emotion strategies that men and women tend to use to regulate their emotions, as some of these strategies are linked to increased somatic symptoms. The interaction between rumination and suppression as well as gender differences in emotion suppression [90] may affect the tendency for women to have higher levels of NA, thereby impacting both rumination and somatic symptoms [77, 91]. The lower use of suppression by men may reduce NA and affect its mediating role on rumination, while greater suppression in women could elevate both NA and rumination. Additionally, previous studies have identified positive affect as a protective factor that may influence the mediating role of NA in women [69, 92].

It should be also note that socialisation factors involving body experience, communication, and pain expression also play a role [82]. Beliefs regarding the unacceptability and fear of expressing negative emotions due to social desirability are influential [93], especially in chronic pain and somatisation studies [92, 94]. Gender has also been a significant predictor of perceived criticism in individuals with chronic pain [73].

Our findings highlight the need for further research examining gender differences in somatisation within a comprehensive biopsychosocial framework. While the present results suggest that ER processes, particularly AR and NA, may partially explain these differences, it is likely that multiple interacting factors are involved.

Our study has a few limitations. It is necessary to consider differences in the populations studied, the measurement tools used, cultural contexts, and the age of the sample as factors that may account for potential differences in the results obtained. A longitudinal design would allow ongoing evaluation [95]. Additionally, somatisation and ER are affected by multiple factors like insecure attachment [96], stress-induced physiological activation [43], or cognitive factors such as symptom interpretation [97], which future research should address, also with a balanced gender sample.

## Conclusions

Given the high prevalence of somatic symptoms and their substantial impact on quality of life, future research should investigate the role of emotional factors in the onset and maintenance of somatisation. Our findings highlight the clinical relevance of interventions aimed at improving ER and reducing persistent NA, as chronic exposure to negative emotional states may increase vulnerability to both psychological and somatic distress. In this regard, evidence-based approaches focused on reducing maladaptive rumination and promoting adaptive emotional strategies may contribute to decreasing symptom severity and improving psychological adjustment.

Furthermore, emotional processes associated with somatic symptoms appear to overlap with mechanisms implicated in chronic pain, supporting the hypothesis of shared pathways related to central sensitisation. These findings have clinical implications. Additional knowledge would help to explore whether this approach can contribute to help prevent the chronification of symptoms, as their taxonomy leads some of them to become structured into chronic syndromes. From a preventive and therapeutic perspective, an integrated approach is encouraged as a form of prevention that focuses on the learning of functional emotional strategies and a reduction of NA, especially in those experiencing somatic symptoms, which could have a protective effect on long-term somatic consequences.

## Availability of Data and Materials

The datasets used and analysed during the current study are available from the corresponding author upon reasonable request.

## References

[1] Henningsen P (2018). Management of somatic symptom disorder. *Dialogues in clinical neuroscience*.

[2] Bourke JH, Langford RM, White PD (2015). The common link between functional somatic syndromes may be central sensitisation. *Journal of psychosomatic research*.

[3] Henningsen P, Zipfel S, Herzog W (2007). Management of functional somatic syndromes. *Lancet (London, England)*.

[4] Serrano-Blanco A, Palao DJ, Luciano JV, Pinto-Meza A, Luján L, Fernández A (2010). Prevalence of mental disorders in primary care: results from the diagnosis and treatment of mental disorders in primary care study (DASMAP). *Social psychiatry and psychiatric epidemiology*.

[5] White PD (2012). Functional somatic syndromes may be either “polysyndromic” or “monosyndromic”. *Journal of psychosomatic research*.

[6] Kroenke K (2003). Patients presenting with somatic complaints: epidemiology, psychiatric co-morbidity and management. *International journal of methods in psychiatric research*.

[7] Gatchel RJ, Peng YB, Peters ML, Fuchs PN, Turk DC (2007). The biopsychosocial approach to chronic pain: scientific advances and future directions. *Psychological bulletin*.

[8] Van den Bergh O, Witthöft M, Petersen S, Brown RJ (2017). Symptoms and the body: taking the inferential leap. *Neuroscience and biobehavioral reviews*.

[9] Afari N, Ahumada SM, Wright LJ, Mostoufi S, Golnari G, Reis V (2014). Psychological trauma and functional somatic syndromes: a systematic review and meta-analysis. *Psychosomatic medicine*.

[10] White PD, Rickards H, Zeman AZJ (2012). Time to end the distinction between mental and neurological illnesses. *BMJ*.

[11] Williams DA (2018). Phenotypic features of central sensitization. *Journal of applied biobehavioral research*.

[12] den Boer C, Dries L, Terluin B, van der Wouden JC, Blankenstein AH, van Wilgen CP (2019). Central sensitization in chronic pain and medically unexplained symptom research: A systematic review of definitions, operationalizations and measurement instruments. *Journal of psychosomatic research*.

[13] Goldenberg DL (2010). Pain/depression dyad: a key to a better understanding and treatment of functional somatic syndromes. *The American journal of medicine*.

[14] Lumley MA, Schubiner H (2019). Emotional awareness and expression therapy for chronic pain: rationale, principles and techniques, evidence, and critical review. *Current rheumatology reports*.

[15] Heim C, Nater UM, Maloney E, Boneva R, Jones JF, Reeves WC (2009). Childhood trauma and risk for chronic fatigue syndrome: association with neuroendocrine dysfunction. *Archives of general psychiatry*.

[16] Dannlowski U, Stuhrmann A, Beutelmann V, Zwanzger P, Lenzen T, Grotegerd D (2012). Limbic scars: long-term consequences of childhood maltreatment revealed by functional and structural magnetic resonance imaging. *Biological psychiatry*.

[17] Davoodi E, Wen A, Dobson KS, Noorbala AA, Mohammadi A, Farahmand Z (2019). Emotion regulation strategies in depression and somatization disorder. *Psychological reports*.

[18] Gilam G, Gross JJ, Wager TD, Keefe FJ, Mackey SC (2020). What is the relationship between pain and emotion? Bridging constructs and communities. *Neuron*.

[19] Rief W, Hennings A, Riemer S, Euteneuer F (2010). Psychobiological differences between depression and somatization. *Journal of psychosomatic research*.

[20] Waller E, Scheidt CE (2006). Somatoform disorders as disorders of affect regulation: a development perspective. *International review of psychiatry (Abingdon, England)*.

[21] Berens S, Schaefert R, Ehrenthal JC, Baumeister D, Eich W, Tesarz J (2021). Different dimensions of affective processing in patients with irritable bowel syndrome: A multicenter cross-sectional study. *Frontiers in psychology*.

[22] Erkic M, Bailer J, Fenske SC, Schmidt SNL, Trojan J, Schröder A (2018). Impaired emotion processing and a reduction in trust in patients with somatic symptom disorder. *Clinical psychology & psychotherapy*.

[23] Lumley MA, Neely LC, Burger AJ (2007). The assessment of alexithymia in medical settings: implications for understanding and treating health problems. *Journal of personality assessment*.

[24] Mewes R, Feneberg AC, Doerr JM, Nater UM (2022). Psychobiological mechanisms in somatic symptom disorder and depressive disorders: an ecological momentary assessment approach. *Psychosomatic medicine*.

[25] Arnold BS, Alpers GW, Süß H, Friedel E, Kosmützky G, Geier A (2008). Affective pain modulation in fibromyalgia, somatoform pain disorder, back pain, and healthy controls. *European journal of pain (London, England)*.

[26] Khosravani V, Samimi Ardestani SM, Alvani A, Amirinezhad A (2020). Alexithymia, empathy, negative affect and physical symptoms in patients with asthma. *Clinical psychology & psychotherapy*.

[27] Schwarz J, Rief W, Radkovsky A, Berking M, Kleinstäuber M (2017). Negative affect as mediator between emotion regulation and medically unexplained symptoms. *Journal of psychosomatic research*.

[28] Geenen R, van Ooijen-van der Linden L, Lumley MA, Bijlsma JW, van Middendorp H (2012). The match–mismatch model of emotion processing styles and emotion regulation strategies in fibromyalgia. *Journal of psychosomatic research*.

[29] Eisma MC, Franzen M, Paauw M, Bleeker A, Aan het Rot M (2022). Rumination, worry and negative and positive affect in prolonged grief: a daily diary study. *Clinical psychology & psychotherapy*.

[30] Gross JJ, Gross JJ (2014). Emotion regulation: Conceptual and empirical foundations. *Handbook of emotion regulation*.

[31] Luyten P, Van Houdenhove B, Lemma A, Target M, Fonagy P (2013). Vulnerability for functional somatic disorders: A contemporary psychodynamic approach. *Journal of Psychotherapy Integration*.

[32] Rasetti R, Mattay VS, Stankevich B, Skjei K, Blasi G, Sambataro F (2010). Modulatory effects of modafinil on neural circuits regulating emotion and cognition. *Neuropsychopharmacology: official publication of the American College of Neuropsychopharmacology*.

[33] Leisman G, Machado C, Melillo R, Mualem R (2012). Intentionality and “free-will” from a neurodevelopmental perspective. *Frontiers in integrative neuroscience*.

[34] Braine A, Georges F (2023). Emotion in action: when emotions meet motor circuits. *Neuroscience and biobehavioral reviews*.

[35] Adachi T, Yamada K, Fujino H, Enomoto K, Shibata M (2022). Associations between anger and chronic primary pain: a systematic review and meta-analysis. *Scandinavian journal of pain*.

[36] Gilam G, Hendler T (2015). Deconstructing anger in the human brain. *Social behavior from rodents to humans*.

[37] Novaco RW, Potegal M, Stemmler G, Spielberger C (2010). Anger and psychopathology. *International handbook of anger*.

[38] Greenwood KA, Thurston R, Rumble M, Waters SJ, Keefe FJ (2003). Anger and persistent pain: current status and future directions. *Pain*.

[39] Galvez-Sánchez CM, Reyes del Paso GA, Duschek S, Montoro CI (2022). The link between fibromyalgia syndrome and anger: a systematic review revealing research gaps. *Journal of clinical medicine*.

[40] Sommer I, Lukic N, Rössler W, Ettlin DA (2019). Measuring anger in patients experiencing chronic pain: a systematic review. *Journal of psychosomatic research*.

[41] Verkuil B, Brosschot JF, Gebhardt WA, Thayer JF, Nyklíček I, Vingerhoets A, Zeelenberg M (2011). Perseverative cognition, psychopathology, and somatic health. *Emotion regulation and well-being*.

[42] Aldao A, Nolen-Hoeksema S (2010). Specificity of cognitive emotion regulation strategies: A transdiagnostic examination. *Behaviour research and therapy*.

[43] Brosschot JF, Gerin W, Thayer JF (2006). The perseverative cognition hypothesis: A review of worry, prolonged stress-related physiological activation, and health. *Journal of psychosomatic research*.

[44] Ottaviani C, Thayer JF, Verkuil B, Lonigro A, Medea B, Couyoumdjian A (2016). The perseverative cognition hypothesis: a review of worry, prolonged stress-related physiological activation, and health. *Psychological Bulletin*.

[45] Ehring T, Watkins ER (2008). Repetitive negative thinking as a transdiagnostic process. *International Journal of Cognitive Therapy*.

[46] Kokonyei G, Szabo E, Kocsel N, Edes A, Eszlari N, Pap D (2016). Rumination in migraine: Mediating effects of brooding and reflection between migraine and psychological distress. *Psychology & health*.

[47] Rogers AH, Bakhshaie J, Ditre JW, Manning K, Mayorga NA, Viana AG (2019). Worry and rumination: explanatory roles in the relation between pain and anxiety and depressive symptoms among college students with pain. *Journal of American college health: J of ACH*.

[48] Schütze R, Rees C, Smith A, Slater H, O’Sullivan P (2020). Metacognition, perseverative thinking, and pain catastrophizing: A moderated-mediation analysis. *European journal of pain (London, England)*.

[49] Ricci A, Bonini S, Continanza M, Turano MT, Puliti EM, Finocchietti A (2016). Worry and anger rumination in fibromyalgia syndrome. *Reumatismo*.

[50] Nolen-Hoeksema S, Wisco BE, Lyubomirsky S (2008). Rethinking rumination. *Perspectives on psychological science: a journal of the Association for Psychological Science*.

[51] Camp J, Blundell E, Smith P, Rimes KA (2025). Emotion regulation differences between gender and sexuality groups: a systematic review and metaanalysis. *Archives of sexual behavior*.

[52] Tamres LK, Janicki D, Helgeson VS (2002). Sex differences in coping behavior: A meta-analytic review and an examination of relative coping. *Personality and Social Psychology Review*.

[53] Johnson JL, Greaves L, Repta R (2009). Better science with sex and gender: facilitating the use of a sex and gender-based analysis in health research. *International journal for equity in health*.

[54] Nolen-Hoeksema S (2012). Emotion regulation and psychopathology: the role of gender. *Annual review of clinical psychology*.

[55] Ballering AV, Lucassen PLBJ, van Tol DG, Goubert L, Bracke P (2025). Sex and gender differences in the public stigmatization of persistent somatic symptoms: a systematic review. *Social science & medicine (1982)*.

[56] Goodman LA (1961). Snowball sampling. *The Annals of Mathematical Statistics*.

[57] Kroenke K, Spitzer RL, Williams JB (2002). The PHQ-15: validity of a new measure for evaluating the severity of somatic symptoms. *Psychosomatic medicine*.

[58] Interian A, Allen LA, Gara MA, Escobar JI, Díaz-Martínez AM (2006). Somatic complaints in primary care: further examining the validity of the Patient Health Questionnaire (PHQ-15). *Psychosomatics*.

[59] Kroenke K, Spitzer RL, Williams JB, Löwe B (2010). The patient health questionnaire somatic, anxiety, and depressive symptom scales: a systematic review. *General hospital psychiatry*.

[60] Watson D, Clark A, Tellegen A (1988). Development and validation of brief measures of positive and negative affect: The PANAS scales. *Journal of personality and social psychology*.

[61] López-Gómez I, Hervás G, Vázquez C (2015). Adaptación de la “Escala de afecto positivo y negativo” (PANAS) en una muestra general española. *Psicol Conductual*.

[62] García-Sancho E, Martín Salguero J, Vasquez EA, Fernández-Berrocal P (2016). Validity and reliability of the Spanish version of the Displaced Aggression Questionnaire. *Psicothema*.

[63] Sandín B, Chorot P, Santed MA, Valiente RM (2002). Estrés y salud: relación de los sucesos vitales y el estrés diario con la sintomatología somática y la enfermedad. *Ansiedad Estrés*.

[64] Batko B (2020). Patient-Centered Care in Psoriatic Arthritis-A Perspective on Inflammation, Disease Activity, and Psychosocial Factors. *Journal of clinical medicine*.

[65] Rogers AH, Gallagher MW, Zvolensky MJ (2023). Intraindividual change in pain tolerance and negative affect over 20 years: findings from the MIDUS study. *Psychology, health & medicine*.

[66] Kirkegaard Thomsen D (2006). The association between rumination and negative affect: A review. *Cognition and Emotion*.

[67] Boyraz G, Efstathiou N (2011). Self-focused attention, meaning, and posttraumatic growth: The mediating role of positive and negative affect for bereaved women. *Journal of Loss and Trauma*.

[68] Van Middendorp H, Lumley MA, Jacobs JWG, van Doornen LJP, Bijlsma JWJ, Geenen R (2008). Emotions and emotional approach and avoidance strategies in fibromyalgia. *Journal of psychosomatic research*.

[69] Watkins ER, Roberts H (2020). Reflecting on rumination: Consequences, causes, mechanisms and treatment of rumination. *Behaviour research and therapy*.

[70] Ray RD, Ochsner KN, Cooper JC, Robertson ER, Gabrieli JDE, Gross JJ (2005). Individual differences in trait rumination and the neural systems supporting cognitive reappraisal. *Cognitive, affective & behavioral neuroscience*.

[71] Burns JW, Gerhart JI, Bruehl S, Peterson KM, Smith DA, Porter LS (2015). Anger arousal and behavioral anger regulation in everyday life among patients with chronic low back pain: Relationships to patient pain and function. *Health psychology: official journal of the Division of Health Psychology, American Psychological Association*.

[72] Van Middendorp H, Lumley MA, Moerbeek M, Jacobs JWG, Bijlsma JWJ, Geenen R (2010). Effects of anger and anger regulation styles on pain in daily life of women with fibromyalgia: A diary study. *European journal of pain (London, England)*.

[73] Mazaheri M, Afshar H, Nikneshan S, Adibi P (2016). Cognitive emotion regulation strategies in patients with functional dyspepsia and healthy controls-A comparative study. *Advanced biomedical research*.

[74] Kiecolt-Glaser JK, McGuire L, Robles TF, Glaser R (2002). Emotions, morbidity, and mortality: new perspectives from psychoneuroimmunology. *Annual review of psychology*.

[75] Lin S, Tan L, Chen X, Liao Z, Li Y, Tang Y (2023). Emotion dysregulation and Internet gaming disorder in young people: mediating effects of negative affect and metacognitions. *Journal of affective disorders*.

[76] Dong HJ, Gerdle B, Bernfort L, Levin LÅ, Dragioti E (2020). Pain catastrophizing in older adults with chronic pain: The mediator effect of mood using a path analysis approach. *Journal of clinical medicine*.

[77] Knowles LM, Ruiz JM, OʼConnor M-F (2019). A systematic review of the association between bereavement and biomarkers of immune function. *Psychosomatic medicine*.

[78] Watkins ER, Mullan E, Wingrove J, Rimes K, Steiner H, Bathurst N (2011). Rumination-focused cognitive-behavioural therapy for residual depression: Phase II randomised controlled trial. *The British journal of psychiatry : the journal of mental science*.

[79] McEvoy PM, Salmon K, Hyett MP, Jose PE, Gutenbrunner C, Bryson K (2019). Repetitive negative thinking as a transdiagnostic predictor of depression and anxiety symptoms in adolescents. *Assessment*.

[80] A-Tjak JG, Davis ML, Morina N, Powers MB, Smits JA, Emmelkamp PM (2015). A meta-analysis of the efficacy of acceptance and commitment therapy for clinically relevant mental and physical health problems. *Psychotherapy and psychosomatics*.

[81] Muñoz-Navarro R, Medrano LA, Limonero JT, González-Blanch C, Moriana JA, Ruiz-Rodríguez P (2022). The mediating role of emotion regulation in transdiagnostic cognitive behavioural therapy for emotional disorders in primary care: Secondary analyses of the PsicAP randomized controlled trial. *Journal of affective disorders*.

[82] Barsky AJ, Peekna HM, Borus JF (2001). Somatic symptom reporting in women and men. *Journal of general internal medicine*.

[83] Brugha TS, Matthews R, Alonso J, Vilagut G, Fouweather T, Bruffaerts R (2013). Gender differences in mental health expectancies in early and midlife in six European countries. *The British journal of psychiatry: the journal of mental science*.

[84] Kudielka BM, Kirschbaum C (2005). Sex differences in HPA axis responses to stress: a review. *Biological psychology*.

[85] Weisenfeld-Hallin Z (2005). Sex differences in pain perception. *Gender medicine*.

[86] Frew AK, Drummond PD (2007). Negative affect, pain and sex: the role of endogenous opioids. *Pain*.

[87] Bruehl S, Burns JW, Chung OY, Ward P, Johnson B (2002). Anger and pain sensitivity in chronic low back pain patients and pain-free controls: the role of endogenous opioids. *Pain*.

[88] Johnson DP, Whisman MA (2013). Gender differences in rumination: A meta-analysis. *Personality and individual differences*.

[89] Kaynar-Yaman G (2026). A review on somatic symptoms and emotion regulation from a cultural perspective. *Nordic journal of psychiatry*.

[90] Zou M, Liu B, Ren L, Mu D, He Y, Yin M (2024). The association between aspects of expressive suppression emotion regulation strategy and rumination traits: a network analysis approach. *BMC psychology*.

[91] Wong WS, Fielding R (2013). Suppression of emotion expression mediates the effects of negative affect on pain catastrophizing: A cross-sectional analysis. *The Clinical journal of pain*.

[92] Sibelli A, Chalder T, Everitt H, Workman P, Bishop FL, Moss-Morris R (2017). The role of high expectations of self and social desirability in emotional processing in individuals with irritable bowel syndrome: a qualitative study. *British journal of health psychology*.

[93] Sydenham M, Beardwood J, Rimes KA (2017). Beliefs about emotions, depression, anxiety and fatigue: a mediational analysis. *Behavioural and cognitive psychotherapy*.

[94] Rimes KA, Ashcroft J, Bryan L, Chalder T (2016). Emotional suppression in chronic fatigue syndrome: experimental study. *Health psychology: official journal of the Division of Health Psychology, American Psychological Association*.

[95] Lamers SM, Bolier L, Westerhof GJ, Smit F, Bohlmeijer ET (2012). The impact of emotional well-being on long-term recovery and survival in physical illness: a meta-analysis. *Journal of behavioral medicine*.

[96] Liu L, Cohen S, Schulz MS, Waldinger RJ (2011). Sources of somatization: Exploring the roles of insecurity in relationships and styles of anger experience and expression. *Social science & medicine (1982)*.

[97] McAndrew LM, Crede M, Maestro K, Slotkin S, Kimber J, Phillips LA (2019). Using the common-sense model to understand health outcomes for medically unexplained symptoms: a meta-analysis. *Health psychology review*.

